# An RNAi-based platform for spatiotemporal control of histone gene expression during animal development

**DOI:** 10.21203/rs.3.rs-6795507/v1

**Published:** 2025-06-04

**Authors:** Oscar M. Arroyo, Mary P. Leatham-Jensen, Daniel J. McKay

**Affiliations:** University of North Carolina at Chapel Hill; University of North Carolina at Chapel Hill; University of North Carolina at Chapel Hill

**Keywords:** histone post-translational modifications, histone mutation

## Abstract

**Background:**

Mutational analysis of histones provides an important means of studying the function of histone post-translational modifications (PTMs) in epigenetic gene regulation. However, several technical challenges have impeded direct tests of histone residue function in metazoans, including the massive abundance of histone gene products, multiple copies of histone genes in the genome, and the necessity of histones for cell viability.

**Results:**

Here, we describe a new experimental approach in *Drosophila* for selective depletion of the replication-dependent histone H3.2. Using short hairpin RNA (shRNA) transgenes, we demonstrate effective depletion of endogenous H3.2 gene expression, which causes defects in cell proliferation and organ development. We further show that a histone replacement transgene, engineered to be insensitive to RNA interference (RNAi) fully rescues shRNA-mediated developmental defects. Last, we demonstrate that this selective depletion platform recapitulates phenotypes caused by histone gene mutation.

**Conclusions:**

We conclude that shRNA-mediated depletion of endogenous histone H3.2, coupled with histone replacement transgenes engineered to be insensitive to RNAi, is an effective experimental approach for studying the role of histone PTMs in animal development.

## Background

During development, a single fertilized egg generates a diversity of cell types with specialized functions to form an adult organism. This cellular diversity arises not from changes to the underlying DNA sequence, but instead through differential gene expression and execution of lineage-specific developmental programs. One essential layer of differential gene regulation comes in the form of epigenetic modifications to chromatin, including post-translational modification of histone proteins.

Histones are the protein cores of nucleosomes, the fundamental repeating unit of chromatin. Nucleosomes are composed of four core histones: H2A, H2B, H3, and H4 [1]. These proteins are encoded by two categories of histone genes. The replication-dependent histone genes are expressed at high levels only during S phase of the cell cycle when large amounts of histone protein are required to package the newly synthesized genome into chromatin [2]. By contrast, the replication-independent histone genes are expressed throughout all cell cycle stages and help to maintain chromatin structure at sites of nucleosome turnover, in addition to their contribution to genome packaging in S phase [3].

Histone post-translational modifications (PTMs) have been directly implicated in regulation of gene expression [4]. In particular, the N-terminal “tails” of histones are subject to a wide array of chemical modifications. Certain modifications, such as trimethylation of histone H3 at lysine residue 27 (H3K27me3), contribute to formation of a repressive chromatin state that silences target gene expression [5]. Other PTMs, such as methylation of histone H3 at lysine residue 4, are correlated with active chromatin states [6]. The co-occurrence of specific histone PTMs in chromatin serves as the basis of the “histone code” hypothesis, which proposes that specific combinations of histone PTMs regulate DNA-templated processes such as transcription, replication, and repair [7]. However, despite strong correlations with gene activity states, the extent to which histone PTMs play a direct role in gene regulation remains unclear [8]. One approach employed to study histone PTM function has been to mutate histone modifying enzymes, such as methyltransferases or acetyltransferases. However, these enzymes often have non-histone substrates and catalytic-independent functions, thus any phenotypes observed may not be due to histone PTM loss alone [8, 9]. An alternative approach is to directly interrogate the phenotypic consequences of histone PTM loss by mutating the histone residues themselves to alternative residues that cannot be modified by enzymes such as methyltransferases or acetylases.

Histone gene manipulation is challenging in most animals because histone genes often exist in many copies that reside at different genomic locations. For instance, the human histone genes are found at three different genomic loci [10]. By contrast, the replication-dependent histone genes of *Drosophila melanogaster* exist at a single genomic locus, referred to as *HisC*, which contains approximately 100 tandemly arrayed copies of a 5kb histone gene repeat unit [11]. Each 5kb repeat contains the four core histone genes plus the linker histone H1 gene. This organization allows for the complete removal of the replication-dependent histone genes with a single genetic deletion, referred to as *ΔHisC* [12]. Homozygous *HisC* deletion results in early embryonic lethality. However, *ΔHisC* lethality can be rescued with transgenes bearing tandem arrays of the 5kb histone gene repeat unit [12, 13]. Combining this type of approach with site-specific mutagenesis of specific histone genes allows for replacement of all the endogenous replication-dependent histone genes in *Drosophila* with mutant versions that cannot be post-translationally modified at specific amino acid residues [13–22].

Although histone gene replacement methods have proven valuable for studying the role of histone PTMs in animal development, limitations still exist [23]. Due to the central role of histones in regulating genome function, many histone mutations are lethal. As a result, histone mutant phenotypes are often investigated through genetic mosaic analysis of homozygous *HisC* deletion clones generated by mitotic recombination. While powerful, recombination-based approaches are limited in spatiotemporal control and by the number of mutant cells that can be analyzed. We sought to determine whether these limitations could be circumvented by employing the GAL4/UAS binary expression system to deplete histone transcripts via short hairpin RNA (shRNA)-induced RNA interference (RNAi). Thousands of GAL4 drivers exist for *D. melanogaster* allowing tight spatiotemporal control of UAS-driven shRNAs targeting histones. We reasoned that shRNA-mediated depletion of endogenous histones could be coupled with transgenic histones engineered to be insensitive to RNAi, thereby allowing for selective expression of transgenic histones in specific tissues and developmental stages. We focus here on the sole replication-dependent histone *H3* gene, *H3.2*.

## Materials & Methods

### Drosophila stocks and genetics

All crosses were maintained on standard corn media (Archon Scientific) at 25°C. The following fly stocks were used: *w; tub-GAL4/CyO act-GFP* (gift of Duronio lab), *w; UAS-GFP* (gift of Matera lab), *w; ey-GAL4/CyO* (BDSC #5535), *w; GMR-GAL4* (BDSC #1104), *prd-GAL4* (BDSC #1947), *yw; act* > *STOP* > *GAL4 UAS-GFP* (BDSC #4411), *nub-GAL4* (BDSC #25754), *CoinFLP-GAL4* (BDSC#58754), *UAS-E(z) RNAi* (BDSC #33659), *ΔHisC*^*Cadillac*^ [23], *H3.2*^*WT − mel*^ [24]. Transgenic lines reported here for the first time were created at BestGene by phiC31 integration into the 86FB attP site (UAS-H3.2-shRNAs) and GenetiVision by phiC31 integration into the VK33 attP site (*H3.2*^*WT − sim*^*, H3.2*^*K27R − sim*^). For adult viability measurements, target genotypes were plotted as a proportion of total progeny. For developmental viability assays, 150 embryos were scored for hatching 48 h after egg laying. Survival of those larvae was subsequently scored. Conventional dissecting scope images were acquired with a Canon EOS Rebel T3i digital camera. To induce mitotic clones, vials were heat-shocked at 37°C for 10 minutes at 48–53 or 72–77 hours after egg laying (h AEL), or 55–60 h AEL for CoinFLP experiments. Larvae were dissected at wandering third instar stage for all imaging of wing and eye-antennal imaginal discs. Pupae were dissected at the pharate adult stage and scored according to the following classes. Class I: Head completely missing to remnants of head cuticle. Class II: Head present with highly reduced to completely missing eyes. Class III: Wildtype head with slightly reduced eyes. Class IV: Wildtype head and eyes. Additional details on crosses are available upon request.

### siRNA design and cloning

The DSIR tool [25] was utilized to generate hairpin sequences against the *D. melanogaster* H3.2 3’-UTR sequence. The following four sequences were selected:

shRNA #1: 5’ – UUAUAGAGUACGCUAGCGCUU – 3’

shRNA #2: 5’ – UUUAUCUGCAAGUUAAUGCCG – 3’

shRNA #3: 5’ – UAUCUGCAAGUUAAUGCCGUG – 3’

shRNA #4: 5’ – UAGCGCUUUAUCUGCAAGUUA – 3’

Oligos were cloned into the VALIUM20 vector [26] following established protocols [27].

siRNA#1 Top Strand:

5’ – CTA GCA GTA AGC GCT AGC GTA CTC TAT AAT AGT TAT ATT CAA GCA TAT TAT AGA GTA CGC TAG CGC TTG CG – 3’

shRNA#1 Bottom Strand:

5’ – AAT TCG CAA GCG CTA GCG TAC TCT ATA ATA TGC TTG AAT ATA ACT ATT ATA GAG TAC GCT AGC GCT TAC TG – 3’

shRNA#2 Top Strand:

5’ – CTA GCA GTC GGC ATT AAC TTG CAG ATA AAT AGT TAT ATT CAA GCA TAT TTA TCT GCA AGT TAA TGC CGG CG – 3’

shRNA#2 Bottom Strand:

5’ – AAT TCG CCG GCA TTA ACT TGC AGA TAA ATA TGC TTG AAT ATA ACT ATT TAT CTG CAA GTT AAT GCC GAC TG – 3’

shRNA#3 Top Strand:

5’ – CTA GCA GTC ACG GCA TTA ACT TGC AGA TAT AGT TAT ATT CAA GCA TAT ATC TGC AAG TTA ATG CCG TGG CG – 3’

shRNA#3 Bottom Strand:

5’ – AAT TCG CCA CGG CAT TAA CTT GCA GAT ATA TGC TTG AAT ATA ACT ATA TCT GCA AGT TAA TGC CGT GAC TG – 3’

shRNA#4 Top Strand:

5’ – CTA GCA GTT AAC TTG CAG ATA AAG CGC TAT AGT TAT ATT CAA GCA TAT AGC GCT TTA TCT GCA AGT TAG CG – 3’

shRNA#4 Bottom Strand:

5’ – AAT TCG CTA ACT TGC AGA TAA AGC GCT ATA TGC TTG AAT ATA ACT ATA GCG CTT TAT CTG CAA GTT AAC TG – 3’

### D. simulans H3.2 3’-UTR and tandem array cloning

The *D. simulans* H3.2 3’-UTR sequence was identified via BLAST [28] and cloned into the 5kb histone gene unit using Q5 mutagenesis (NEB). Tandem arrays containing twelve copies of the histone gene unit were generated as previously described [29].

### Sequence comparisons of H3.2 paralogs

The H3.2 gene copy number was determined via BLAST using the *D. melanogaster* H3.2 reference and the complete assembly of the histone locus [30]. Sequence identity was analyzed using MATLAB (Mathworks) Bioinformatic Toolbox.

### Immunofluorescence

Standard immunostaining protocols were performed for embryos, wing imaginal discs, and eye-antennal imaginal discs. Primary antibodies and concentrations are as follows: mouse anti-GFP (Abcam, cat# ab1218) 1:500, rabbit anti-H3 (Abcam, cat# ab1791) 1:1000, mouse anti-Ubx (Developmental Studies Hybridoma Bank, cat# FP3.38) 1:30, rabbit anti-H3K27me3 (Cell Signaling Technology, cat# 9733) 1:500. Secondary antibodies used at 1:1000: Alexa Fluor 488 (Thermo Fisher Scientific, cat # A32723), Alexa Fluor 555 (Thermo Fisher Scientific, cat # A32732), Alexa Fluor 555 (Thermo Fisher Scientific, cat # A21424), Alexa Fluor 647 (Thermo Fisher Scientific, cat# A21245). Confocal images were acquired using a Leica SP8 and processed in ImageJ. Uniform adjustments were made to brightness and/or contrast. For image quantification, signal intensity in regions of interest was determined using ImageJ and analyzed using MATLAB. Thresholding of GFP signal was used to determine GFP + regions. After background subtraction, the mean signal intensity for each region was calculated for each embryo and plotted.

### Scanning electron microscopy

Scanning electron microscopy (SEM) images of newly eclosed (≤ 48 h) and dissected pharate adults were acquired with a Hitachi TM4000Plus tabletop SEM microscope. Dissected pharate adults were imaged at 10 kV and 100× magnification while GMR-GAL4 flies were imaged at 15 kV and 200x magnification.

## Results

### An RNAi Based Platform for Manipulating Histone Gene Expression

**Figure 1:**
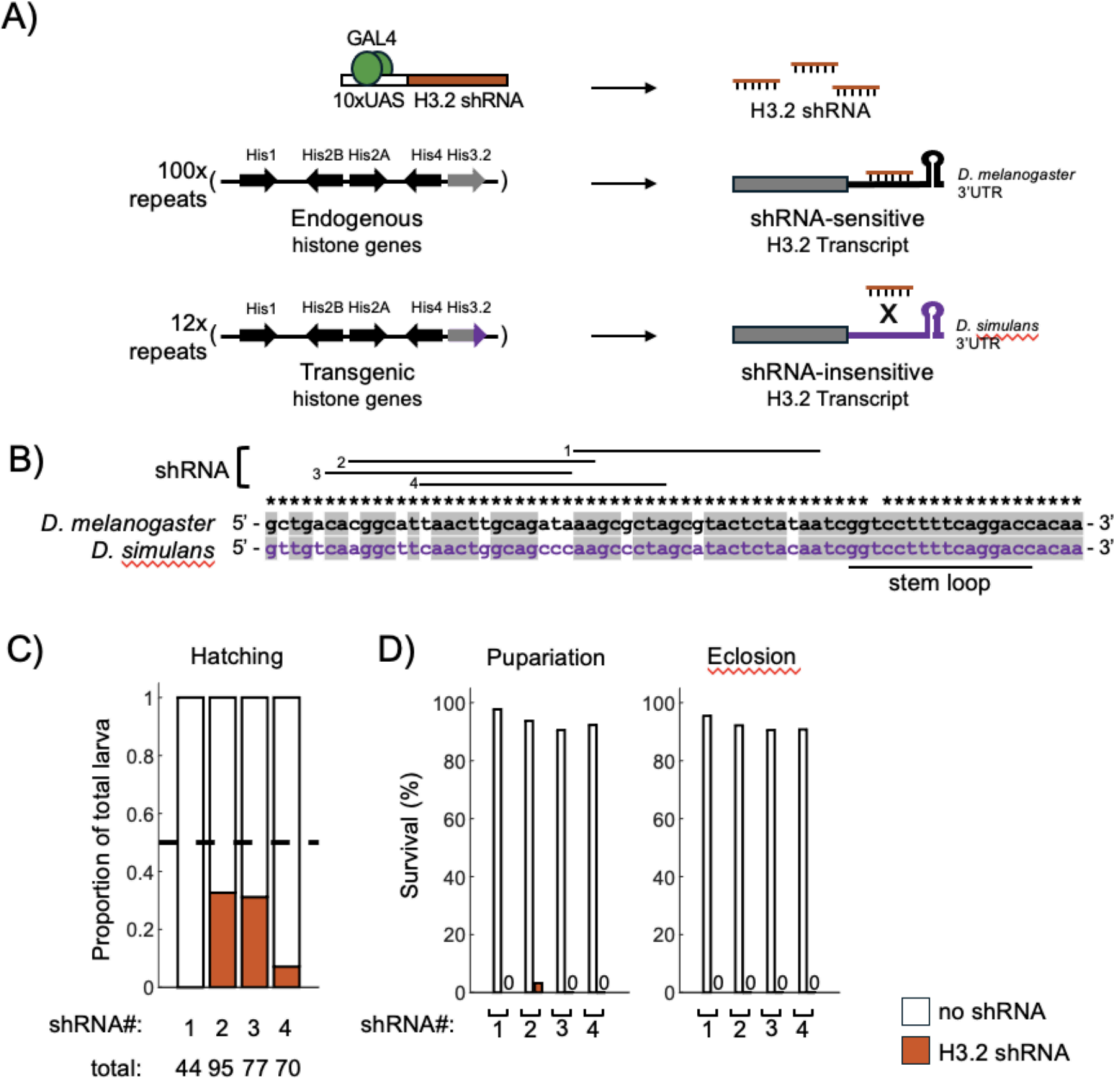
A system for selective depletion of endogenous histone H3.2 transcripts (A) Schematics of H3.2 shRNA transgene (top), endogenous histone genes (middle), and transgenic histone genes with *D. simulans H3.2* 3’-UTR indicated in purple (bottom). (B) DNA sequence corresponding to the *H3.2* 3’-UTR from *D. melanogaster* and *D. simulans*. Asterisks indicate nucleotide identity across all *D. melanogaster H3.2* paralogs. Shading indicates nucleotide identity between *D. melanogaster* and *D. simulans*. Horizontal lines indicate shRNA target sequences for each hairpin. (C) Stacked bar charts depicting the proportion of larvae that express the H3.2 shRNA vs control siblings that do not express the shRNA. The expected proportion is indicated with a dashed line. (D) Bar charts depicting the survival of larvae from C to pupal stages (pupariation) and adulthood (eclosion).

For an RNAi-based approach to be effective at depleting endogenous histone gene expression (Fig. 1A), the shRNA must successfully target transcripts produced by each of the ~ 100 copies of the endogenous *H3.2* gene. Any variation in shRNA target sequence across the tandem array would reduce efficacy of *H3.2* depletion. To investigate sequence composition across histone *H3.2* gene repeats, we examined a recent long-read sequence assembly of the entire *HisC* locus [30]. Bioinformatic analysis identified 108 copies of the endogenous histone *H3.2* gene. All but one copy is predicted to encode identical full-length H3.2 proteins. One gene copy is truncated and predicted to be non-functional; this gene was excluded from further analysis. Alignment of sequences corresponding to the *H3.2* 3’-UTR revealed 100% sequence identity for 103/107 gene copies, whereas 4/107 gene copies share the same single nucleotide polymorphism (Fig. 1B, **Supplemental Fig. 1**). Sequence identity across the 3’-UTRs is even greater than that observed for the *H3.2* coding sequence, for which 92/107 gene copies are identical and 15/107 have synonymous polymorphisms (**Supplemental Fig. 1**). The high degree of sequence identity across all *H3.2* gene copies makes the 3’-UTR a favorable target for shRNA-mediated depletion. Targeting the 3’-UTR also provides a straightforward means of generating H3.2 transcripts insensitive to shRNA-mediated depletion by swapping the *D. melanogaster* sequence with the 3’-UTR from the closely related species, *D. simulans*. Importantly, the sequence of the stem loop, a *cis*-element located within the 3’-UTR required for histone mRNA processing, is conserved between the two species (Fig. 1B), suggesting that the *D. simulans* H3.2 3’-UTR may functionally replace the *D. melanogaster* 3’-UTR. Furthermore, sequence divergence upstream of the stem loop allows for design of shRNA constructs whose products would hybridize perfectly with *D. melanogaster* histone H3.2 3’-UTRs, leading to their degradation, whereas mismatches with *D. simulans* 3’-UTRs would render them insensitive to shRNA-mediated degradation, thereby enabling selective depletion of endogenous H3.2 (Fig. 1B).

We designed four GAL4/UAS-driven shRNA constructs targeting overlapping regions in the *D. melanogaster* H3.2 3’-UTR (Fig. 1B) and generated independent transgenic lines via site-specific integration. We also generated a histone replacement transgene with 12 tandem repeats encoding wild-type histone proteins in which the sequence corresponding to the *D. melanogaster H3.2* 3’-UTR was replaced by the *D. simulans* sequence (hereafter, *H3.2*^*WT − sim*^). Below, we test whether these elements allow for spatiotemporal control of histone gene expression via selective depletion. We first determine whether these shRNAs can deplete endogenous histone H3.2 expression and generate developmental phenotypes consistent with histone loss-of-function. Next, we examine whether shRNA-insensitive replacement transgenes encoding wild-type histone H3.2 rescue endogenous histone gene deletion and shRNA-induced phenotypes. Last, we test whether shRNA-insensitive replacement transgenes encoding mutant H3.2 proteins result in histone mutant phenotypes upon shRNA expression.

### Histone H3.2-shRNA expression causes cell proliferation defects due to reduced H3 protein

Diploid *D. melanogaster* cells contain approximately 200 histone *H3.2* gene copies, and their mRNA and protein gene products are among the most abundant in the cell [31, 32]. Due to this abundance, we first sought to determine whether shRNAs targeting endogenous H3.2 would be capable of generating developmental phenotypes associated with histone depletion. Homozygous deletion of the *HisC* locus results in embryonic lethality due to cell cycle arrest before the onset of mitosis during the 15th nuclear cycle [12, 33]. Prior to this stage, maternally supplied histone gene products are used to package the newly replicated genome. However, maternal histone mRNAs are degraded at the end of cycle 14 [34], and the absence of zygotically encoded gene products in *DHisC* mutants leads to a reduction in the pool of histone proteins and cell cycle arrest [35]. In addition to *H3.2*, *DHisC* mutants lack all other replication-dependent histone genes (*H4, H2A, H2B, H1*). Whereas viability of animals lacking only histone *H3.2* has not been reported, clones of cells lacking *H3.2* were reported to exhibit severe growth defects [36], presumably due to incomplete nucleosome assembly during DNA replication. To determine the consequences of histone H3.2 shRNA expression during development, we first used a ubiquitous GAL4 driver that turns on early in embryogenesis. We found that expression of each of the four shRNAs results in lethality. Few to no embryos ubiquitously expressing shRNA #1 or shRNA #4 hatched into larvae, whereas approximately 30% of embryos expressing shRNA #2 or shRNA #3 hatched (Fig. 1C, D). However, nearly all shRNA-expressing larvae died, with only a single larva progressing to the pupal stage. Importantly, we recovered zero shRNA-expressing adults (Fig. 1D). Thus, expression of each H3.2 shRNA is deleterious for development.

**Figure 2:**
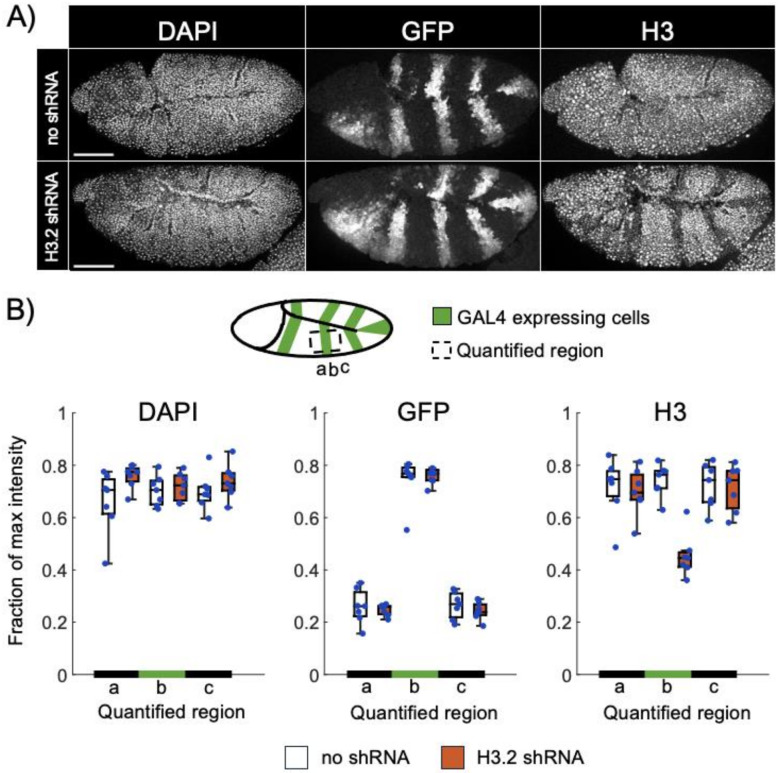
Expression of H3.2 shRNA causes depletion of H3 protein. (A) Confocal images of embryos stained for DAPI (left), GFP (middle), and total H3 (right) for no shRNA control (top) and H3.2 shRNA-expressing embryos (bottom). Scale bars – 100 micrometers. (B) Representation of *prd-GAL4* expression pattern. Signal was quantified for the region within the dashed box and indicated below each box plot (a, b, c). Box plots of normalized max intensity for DAPI (left), GFP (middle), and H3 (right) signal. The median is indicated by the horizontal line, and individual data points are shown with blue dots.

To determine the impact of shRNA expression on histone H3 protein levels, we performed immunofluorescence in early embryos using the *prd-GAL4* driver, which is active in segmentally repeated stripes in the epidermis (Fig. 2A). Total histone H3 levels were evaluated approximately 1.5-hours after the onset of *prd-GAL4* expression, a time in development when epidermal cells are actively cycling [35, 37]. In contrast to control genotypes, we observed a reduction in histone H3 protein in shRNA-expressing cells relative to non-expressing cells (Fig. 2B). Image quantification indicates a 42% decrease in histone H3 signal in shRNA-expressing regions relative to control regions (Fig. 2B). This decrease in total H3 protein levels approaches the maximum expected reduction of 50% due to a single S phase occurring after the onset of *prd-GAL4* activation [35], and it is not expected that shRNA-mediated depletion would impact the levels of H3 protein already present in chromatin before *prd-GAL4* activation. The observed reduction in total H3 protein levels also indicates that the replication-independent H3.3 genes do not substantially compensate for H3.2 depletion. We also note that we did not detect a decrease in DAPI levels in shRNA-expressing cells relative to control cells (Fig. 2B), consistent with prior studies of *DHisC* embryos that determined DNA replication continues in epidermal cells at this stage of embryogenesis even in the absence of all replication-dependent histone genes [35]. We conclude that shRNA expression quickly and effectively reduces histone H3.2 protein levels despite the high copy number of *H3.2* genes.

**Figure 3:**
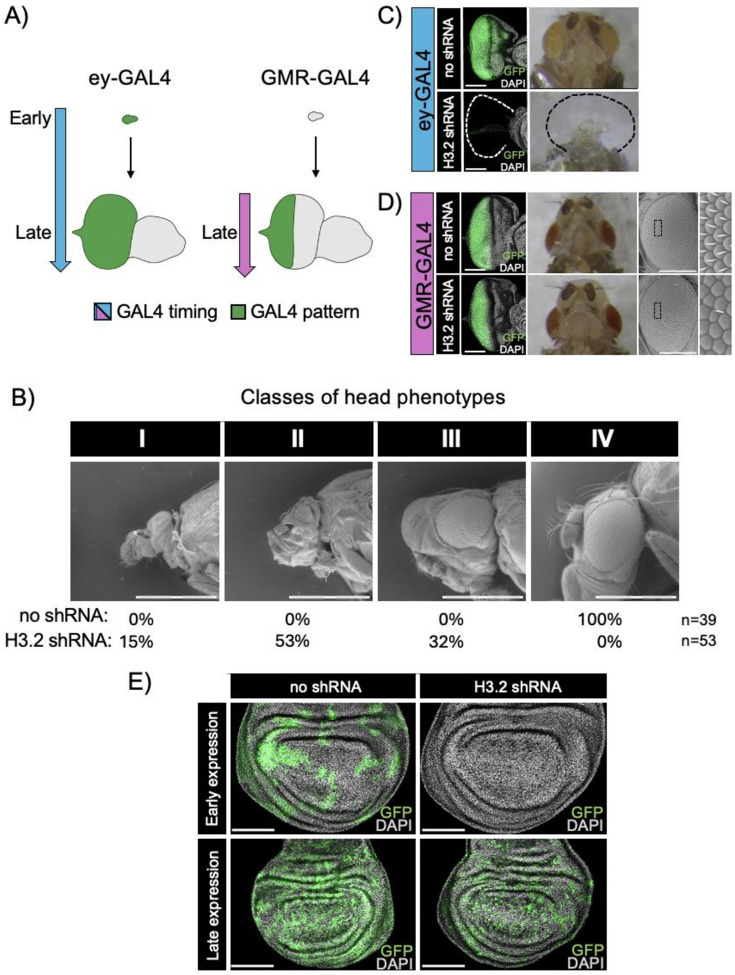
H3.2 shRNA expression causes proliferation defects. (A) Cartoon of eye-antennal disc development depicting spatiotemporal patterns of *ey-GAL4* and *GMR-GAL4* expression. Temporal pattern is indicated by arrows. Spatial pattern is indicated by green shading. (B) Scanning electron micrographs of pharate adult heads. Representative images of four phenotypic classes of decreasing severity are presented. The percentage of control and shRNA-expressing adults belonging to each class is indicated. Scale bars – 500 micrometers. (C) Confocal images of wandering 3^rd^ instar eye-antennal discs (left). Control eye-antennal disc depicting *ey-GAL4* driven GFP expression (top). *ey-GAL4* H3.2 shRNA expressing eye-antennal disc (bottom). Stereomicrograph of pharate adult heads from the corresponding genotypes (right). Scale bars – 100 micrometers. (D) Confocal images of wandering 3^rd^ instar eye-antennal discs (left). Control eye-antennal disc depicting *GMR-GAL4* driven GFP expression (top). *GMR-GAL4* H3.2 shRNA expressing eye-antennal disc (bottom). Stereomicrograph of pharate adult heads from the corresponding genotypes (middle). Scale bars – 100 micrometers. Scanning electron micrographs of adult eyes from the corresponding genotypes (right). Scale bars – 200 micrometers. (E) Confocal images of 3^rd^ instar wing imaginal disc clones expressing GFP (left), or GFP and H3.2 shRNA (right). Scale bars – 100 micrometers.

To examine the consequences of shRNA expression on organ development, we turned to the *Drosophila* eye, which has been widely used as a model to understand developmental control of cell proliferation [38, 39]. The adult compound eye is derived from a larval tissue known as the eye-antennal imaginal disc, which along with the eye and antenna, produces most of the external structures of the adult head [40]. Development of the eye-antennal imaginal disc is characterized by a proliferative phase in which cells asynchronously progress through the cell cycle, followed by a post-proliferative phase when most of the cells exit the cell cycle to establish a terminal fate. Within the eye, these two phases can be interrogated separately using the *ey-GAL4* driver [41], which is active early in eye development during the proliferative phase, and the *GMR-GAL4* driver [42], which is active later during the last round of cell division in the eye and in the post-proliferative phase (Fig. 3A).

H3.2 shRNA expression under control of *ey-GAL4* resulted in a severe reduction in viability. H3.2 shRNA-expressing animals represented no more than 10% of the observed progeny, which is a significant reduction relative to the expected mendelian ratio of 50% (**Supplemental Fig. 2A**). Notably, all surviving adults exhibited defects in eye and head morphology, indicating that phenotypes caused by H3.2 shRNA expression are fully penetrant. Closer inspection of pharate adults that failed to eclose from their pupal cases using scanning electron microscopy (SEM) revealed that shRNA expression under control of *ey-GAL4* resulted in extensive loss of eye tissue and other head structures, including animals that completely lacked heads (Fig. 3B). Consistent with these observations, shRNA-expressing 3rd instar eye-antennal imaginal discs exhibited highly reduced to absent eye tissue in the most extreme cases (Fig. 3C). Interestingly, we observed increased severity of phenotypes in shRNA-expressing males relative to females. For instance, none of the surviving adults were male (**Supplemental Fig. 2A**), and pharate adult males exhibited more severe defects in eye and head morphology relative to females (**Supplemental Fig. 2B**). The cause for the increased phenotypic severity in males is unclear; however, the vector we used for shRNA expression has been reported to have stronger effects in males than in females [43], consistent with our observations.

Loss of eye and head tissue upon shRNA expression during the proliferative stage of eye development suggests that H3.2 protein was sufficiently depleted to cause a proliferation defect. An alternative explanation is that tissue loss was due to cytotoxicity or off-target effects of shRNA expression. To investigate this possibility, we drove shRNA expression in eye imaginal discs using the later-acting *GMR-GAL4*. Replication-dependent histones are only expressed during S phase [2]. Therefore, we reasoned that if the observed phenotypes were independent of histone H3.2 depletion, then shRNA expression during the post-proliferative phase would also result in tissue loss. Instead, we observed that *GMR-GAL4* driven H3.2 shRNA expression resulted in no detectable changes to eye imaginal disc morphology (Fig. 3D). Moreover, adult eyes examined five days after *GMR-GAL4* activation appeared normal under conventional dissecting microscopes (Fig. 3D). However, closer examination of shRNA-expressing adults using SEM revealed subtle changes to eye morphology relative to control *GMR-GAL4* > *UAS-GFP* flies, including disruptions to the hexagonal array of ommatidia and missing interommatidial bristles (Fig. 3D, **inset**). Similar rough eye phenotypes and loss of interommatidial bristles have previously been reported when the *GMR* promoter was used to drive expression of the cell cycle inhibitor, p21 [44, 45]. Gene expression driven by *GMR* can disrupt the second mitotic wave, reducing the number of precursor cells available for terminal differentiation in the eye, leading to rough eye phenotypes [44]. Likewise, interommatidial bristles are produced by two cell divisions that take place during pupal stages of eye development, after *GMR-GAL4* becomes active [45]. Thus, subtle rough eye phenotypes and interommatidial bristle loss is expected if histone H3.2 shRNA expression disrupts cell proliferation. Together, these findings indicate that loss of tissue upon histone H3.2 shRNA expression is not due to cytotoxicity or off-target effects. Instead, these findings suggest that H3.2 shRNA expression results in a cell proliferation defect due to depletion of histone H3.2 protein.

In a final set of experiments, we examined the consequences of H3.2 shRNA expression with increased temporal and cellular resolution by performing mosaic analysis in developing wing imaginal discs. Clones of cells expressing GFP alone or GFP plus histone H3.2 shRNA were generated via *FLP/FRT* mediated excision of a transcriptional stop cassette located between the constitutively active *Actin 5C* promoter and GAL4 coding sequence (ie. “FLP-out” clones) [46]. Induction of FLP 67–72 hours prior to dissection yielded large GFP-positive clones located throughout the wing imaginal disc epithelium in the control genotype (n = 10 wings) (Fig. 3E). By contrast, GFP-positive clones were completely absent from the H3.2 shRNA-expressing genotype (n = 21 wings), consistent with elimination of shRNA-expressing cells caused by a growth disadvantage relative to neighboring wild-type cells [47]. Decreasing the duration of shRNA expression by decreasing the length of time between FLP induction and dissection led to the recovery of H3.2 shRNA-expressing clones (n = 14 wings), which nevertheless appeared fewer in number relative to clones made in control discs (Fig. 3E).

We next attempted to compare the growth of control clones and H3.2 shRNA-expressing clones in the same tissue by employing the CoinFLP system, which allows for clonal expression of either GAL4 (shRNA) or LexGAD (control) via mutually exclusive FLP-induced recombination [48]. Distinct fluorescent reporters distinguish GAL4-expressing clones (CD8::RFP) from LexGAD-expressing clones (CD8::GFP). However, comparison of clone sizes between control and shRNA-expressing cells was not possible due to the recovery of very few control LexGAD clones for unknown reasons (**Supplemental Fig. 3**). Nevertheless, comparison of GAL4-expressing clones from shRNA-containing and shRNA-lacking wing imaginal discs yielded similar results as the FLP-out experiments described above in that shRNA-expressing clones were smaller than shRNA-lacking clones (**Supplemental Fig. 3**). Closer inspection revealed that shRNA-expressing clones appeared fragmented relative to shRNA-lacking clones (**Supplemental Fig. 3, inset**), and fragments of fluorescently tagged membrane (CD8::RFP) were often detected at the basal surface of the wing imaginal disc. These observations are consistent with elimination of shRNA-expressing cells from the epithelium due to a growth defect [49]. Altogether, these findings demonstrate that H3.2 shRNA expression leads to a proliferation defect, likely due to reduced H3.2 protein.

### The 3’-UTR of D. simulans H3.2 rescues histone locus deletion in D. melanogaster

**Figure 4:**
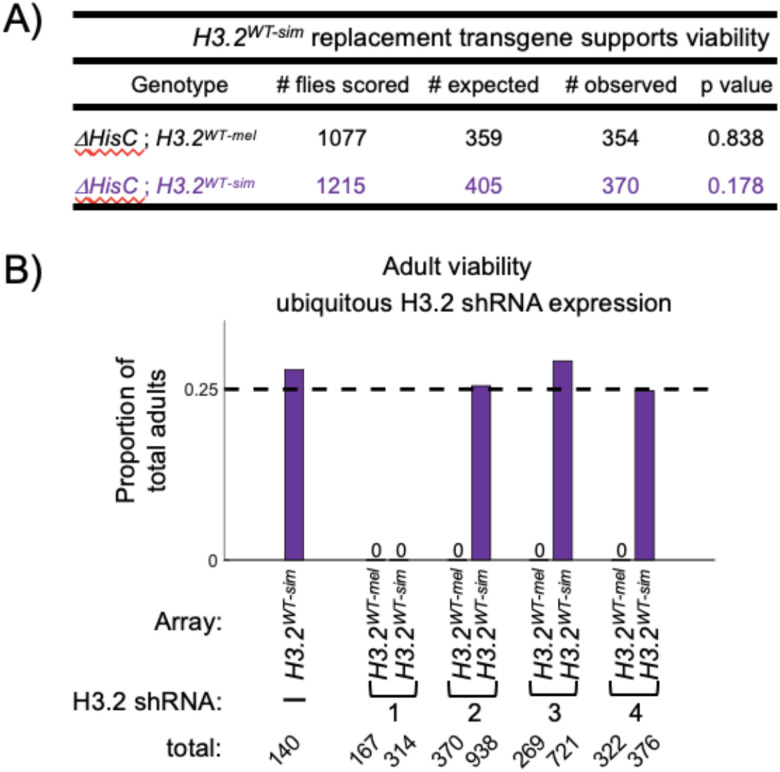
*H3.2*^*WT-sim*^ replacement transgene supports wildtype viability and rescues ubiquitous H3.2 depletion in D. melanogaster. (A) Table of viability for *H3.2*^*WT-mel*^ and *H3.2*^*WT-sim*^ replacement transgenes in *D. melanogaster*. The p values for the chi-square test are shown. (B) Bar chart depicting the proportion of total adults with ubiquitous H3.2 shRNA expression that are female for *H3.2*^*WT-mel*^ and *H3.2*^*WT-sim*^ replacement transgenes. The expected proportion is indicated with a dashed line.

Replication-dependent histone transcripts are the only non-polyadenylated mRNAs in metazoans [50]. Instead of a poly-A tail, they terminate in a 3’ stem loop that is responsible for proper transcript processing, translation, and degradation [51]. As noted above, the histone H3.2 stem loop sequence is identical between *D. melanogaster* and *D. simulans*. However, sequence composition varies upstream of the stem loop, raising the question whether the *D. simulans* 3’-UTR would function in *D. melanogaster*. To test the functionality of the *D. simulans* H3.2 3’-UTR, we asked whether *H3.2*^*WT − sim*^ could support development in the absence of the endogenous replication-dependent histone genes by generating a transgenic line bearing twelve tandem histone gene repeats integrated at the same genomic locus as the existing *H3.2*^*WT − mel*^ transgene [24]. We found that one copy of the *H3.2*^*WT − sim*^ transgene rescued homozygous *HisC* deletion. More importantly, *H3.2*^*WT − sim*^ adults eclosed at the expected mendelian ratio, similar to the *H3.2*^*WT − mel*^ control genotype (Fig. 4A). We conclude that the 3’-UTR of *D. simulans* H3.2 supports normal histone H3.2 expression in *D. melanogaster*.

### The 3’-UTR of D. simulans is insensitive to shRNA-mediated H3.2 depletion in D. melanogaster

We next sought to determine whether mismatches in the *D. simulans* 3’-UTR sequence render it insensitive to shRNA-mediated H3.2 depletion. First, we crossed *H3.2*^*WT − sim*^ flies bearing a ubiquitous GAL4 driver to each H3.2 shRNA and scored adult viability (Fig. 4B). As before, these experiments were conducted in the presence of the endogenous replication-dependent histone genes. To account for the extra twelve *H3.2* gene copies contributed by the histone replacement transgene, we performed a control cross using *H3.2*^*WT − mel*^. Whereas *H3.2*^*WT − mel*^ crosses produced zero adult progeny for all four shRNAs, crosses with the *H3.2*^*WT − sim*^ replacement transgene produced adult progeny for three out of the four shRNAs (Fig. 4B). No adults were obtained for shRNA#1, suggesting that it may have off-target effects, or it may deplete *H3.2*^*WT − sim*^ expression in addition to endogenous *H3.2*. Interestingly, we observed differences in the numbers of shRNA-expressing adult males and females rescued by *H3.2*^*WT − sim*^. Whereas the number of shRNA-expressing *H3.2*^*WT − sim*^ females matched the number of non-shRNA expressing control *H3.2*^*WT − sim*^ females, fewer than expected shRNA-expressing *H3.2*^*WT − sim*^ males were recovered (**Supplemental Fig. 4A**). Although the basis for this sex difference is unclear, this finding is consistent with the increased severity of H3.2 shRNA-mediated defects observed in males lacking a histone replacement transgene, as described above (**Supplemental Fig. 2A**).

**Figure 5:**
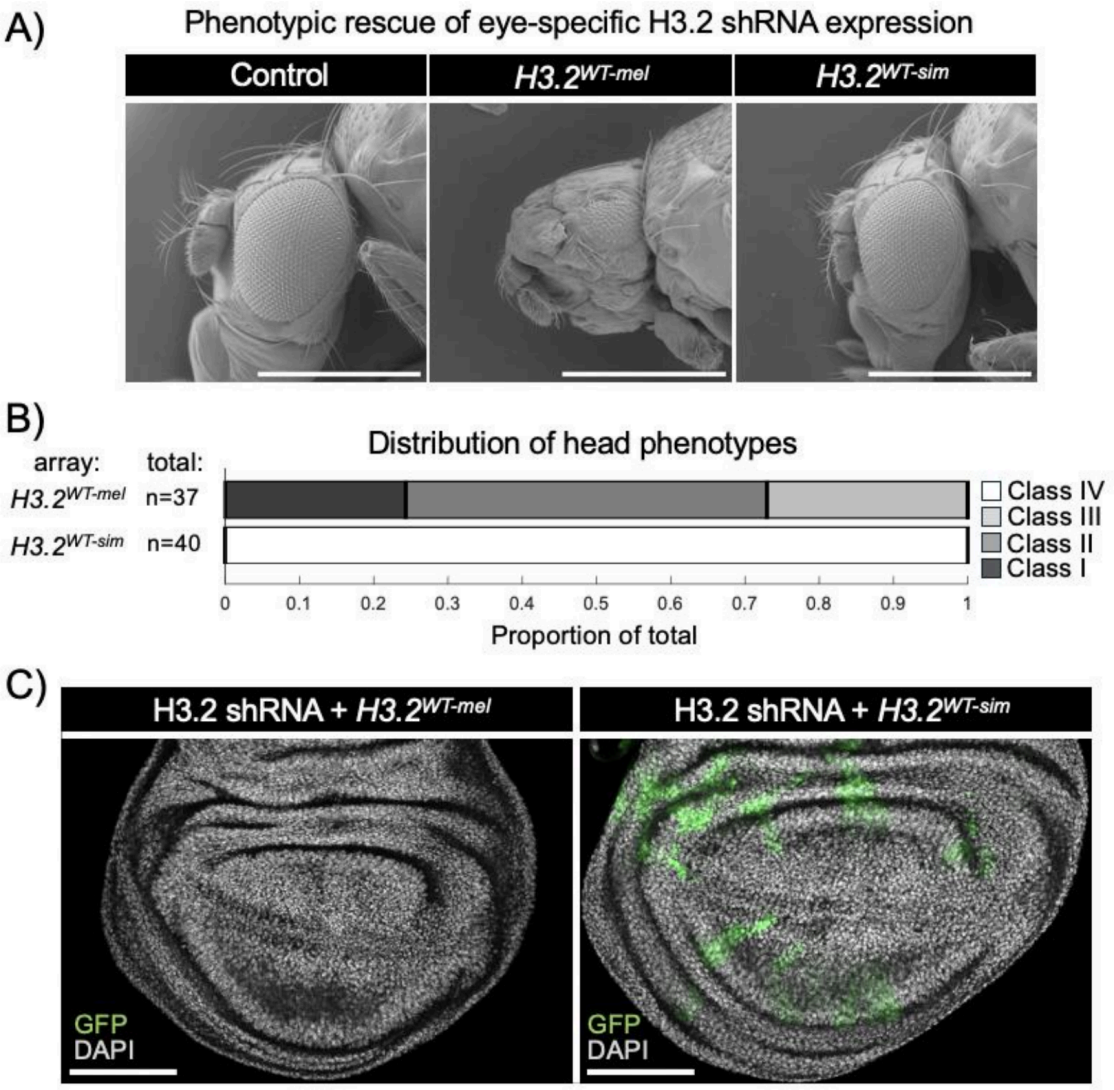
Phenotypic rescue of H3.2 shRNA expression in eye-antennal and wing imaginal discs in *D. melanogaster*. (A) Scanning electron micrographs of pharate adult heads. Scale bars – 100 micrometers. (B) Stacked bar chart depicting the proportion of pharate adults in four phenotypic classes of head defects. shRNA expression driven by *ey-GAL4*. (C) Confocal images of 3^rd^ instar wing imaginal discs. Clones expressing H3.2 shRNA are marked with GFP with *H3.2*^*WT-mel*^ (left) and *H3.2*^*WT-sim*^ replacement transgenes (right). Scale bars – 100 micrometers.

We next tested the ability of the *H3.2*^*WT − sim*^ replacement transgene to rescue shRNA-mediated defects in the developing eye. Similar to our findings with ubiquitous shRNA expression, the *H3.2*^*WT − sim*^ replacement transgene rescued adult viability when H3.2 shRNAs were expressed under control of *ey-GAL4*, whereas the *H3.2*^*WT − mel*^ replacement transgene did not rescue adult viability (**Supplemental Fig. 4B**). Moreover, the *H3.2*^*WT − sim*^ replacement transgene fully rescued shRNA-mediated defects in head and eye development (Fig. 5A–B), whereas *H3.2*^*WT − mel*^ replacement flies exhibited the same distribution of head and eye defects caused by *ey-GAL4* driven shRNA expression in the absence of a histone replacement transgene (Fig. 3B). Surprisingly, we observed no differences in male and female viability in *H3.2*^*WT − sim*^ flies expressing shRNAs under control of *ey-GAL4* (**Supplemental Fig. 4C**), unlike our findings when *ey-GAL4* was used to drive shRNA expression without a histone replacement transgene (**Supplemental Fig. 2A**). We also observed that the *H3.2*^*WT − sim*^ replacement transgene rescues viability of shRNA#1 driven by *ey-GAL4* (**Supplemental Fig. 4B**), unlike our findings with ubiquitous expression of shRNA#1 (Fig. 4A), indicating that some of the phenotypes caused by this shRNA are cell-type specific. Last, we examined the ability of the *H3.2*^*WT − sim*^ replacement transgene to rescue growth of shRNA-expressing clones in developing wing imaginal discs. Similar to results obtained in the absence of a histone replacement transgene, we failed to obtain shRNA-expressing clones in the presence of the *H3.2*^*WT − mel*^ replacement transgene (Fig. 5C). However, we observed large shRNA-expressing clones in the presence of the *H3.2*^*WT − sim*^ replacement transgene (Fig. 5C). Together, these findings indicate that the *D. simulans* H3.2 3’-UTR is insensitive to shRNA-mediated knockdown, and it supports normal viability and cell proliferation despite depletion of endogenous H3.2 expression.

### Selective depletion of endogenous H3.2 with a mutant histone replacement transgene results in Polycomb target gene derepression

**Figure 6:**
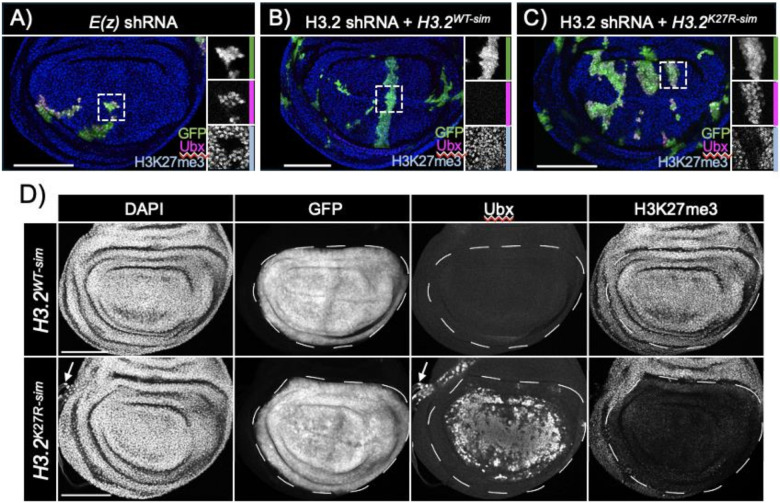
Selective depletion of endogenous H3.2 causes Ubx derepression with *H3.2*^*K27R-sim*^ replacement transgene. (A, B, C) Confocal images of 3^rd^ instar wing imaginal discs stained for H3K27me3 (blue) and Ubx (magenta). Clones expressing shRNA are marked with GFP (green) for: (A) E(z) shRNA, (B) H3.2 shRNA with *H3.2*^*WT-sim*^ replacement transgene, and (C) H3.2 shRNA with *H3.2*^*K27R-sim*^ replacement transgene. (D) Confocal images of 3^rd^ instar wing imaginal discs with H3.2 shRNA driven by nub-GAL4 stained for DAPI, GFP, Ubx, and H3K27me3. Control *H3.2*^*WT-sim*^ replacement transgene (top), *H3.2*^*K27R-sim*^ replacement histone array (bottom). Arrow marks tracheal tissue that endogenously expresses Ubx. Scale bars – 100 micrometers.

Histone gene replacement approaches allow for investigation of epigenetic mechanisms in animals by combining genetic deletion of the endogenous replication-dependent histone genes with replacement transgenes encoding mutant histones [23]. To determine whether shRNA-mediated selective depletion of endogenous histone gene expression can be used to study the consequences of histone residue mutation, we generated an shRNA-insensitive histone replacement transgene that encodes a mutant H3.2 protein. We focused on histone H3.2 lysine 27 (H3.2K27), whose methylation is required for silencing of *Polycomb* target genes. Mutation of H3.2K27 to a non-modifiable arginine residue (H3.2K27R) results in derepression of Polycomb target genes, such as the Hox gene *Ultrabithorax (Ubx)* in developing wings [13, 14, 52]. We performed a series of clonal analyses to determine the impact of shRNA expression on *Ubx* expression in the main epithelium of 3rd instar wing imaginal discs. Consistent with expectations, clones expressing shRNAs targeting Enhancer of zeste (E(z)), the sole methyltransferase responsible for H3K27 methylation in *D. melanogaster*, exhibited derepression of *Ubx*, and a decrease in H3K27me3 levels (Fig. 6A). Next, we made clones of cells expressing histone H3.2 shRNAs in the presence of control *H3.2*^*WT − sim*^ replacement transgenes and observed no derepression of *Ubx* or change in H3K27me3 levels (Fig. 6B). By contrast, H3.2 shRNA-expressing clones in which the insensitive histone replacement transgene encodes mutant H3.2K27 (*H3.2*^*K27R − sim*^) exhibited strong *Ubx* derepression and decreased H3K27me3 levels (Fig. 6C). Thus, shRNA-mediated knockdown of endogenous histone H3.2 in the presence shRNA-insensitive H3.2K27R replacement histones reduces the abundance of wild-type histone below the minimum threshold necessary for maintaining repression of Polycomb target genes. In a further test of this platform’s ability to selectively express mutant histones with high spatiotemporal precision, we expressed histone H3.2 shRNA under control of *nubbin-GAL4*, which is active in the developing wing imaginal disc pouch [53]. Whereas the control *H3.2*^*WT − sim*^ replacement transgene supported normal H3K27me3 levels and *Ubx* repression, the *H3.2*^*K27R − sim*^ replacement transgene resulted in decreased H3K27me3 levels and robust *Ubx* derepression (Fig. 6D). We note that *Ubx* derepression was weakest in the peripheral regions of the wing pouch. *Nub* gene expression is thought to be activated in these more proximal cells later in wing disc development relative to more distal cells in the center of the pouch [54]. As a result, these cells likely express H3.2 shRNA under control of *nub-GAL4* for a shorter duration, limiting the time and number of cell divisions during which endogenous H3.2 expression can be depleted. Consistent with this interpretation, H3K27me3 levels are higher in these peripheral, shRNA-expressing, non-*Ubx*-derepressing cells (Fig. 6D). Altogether, we conclude that sufficient mutant histone is incorporated into chromatin to trigger Polycomb target gene derepression due to shRNA-mediated endogenous histone H3.2 depletion. More generally, these findings demonstrate that selective histone depletion can be used to generate mutant tissues, expanding the set of experimental approaches that can be used to examine histone mutant phenotypes.

## Discussion

Technical challenges associated with mutating histone genes *in vivo* has impeded the study of histone function in animal models. As a result, our understanding of metazoan histone PTM function has been largely inferred from indirect evidence obtained via mutation of the genes encoding chromatin binding and modifying enzymes. We describe here a new experimental approach that relies on selective depletion of endogenous histone gene expression coupled with histone replacement transgenes engineered to be insensitive to shRNA targeting. Expression of shRNAs effectively depletes endogenous histone H3.2. Replacement transgenes encoding insensitive wild-type histone H3.2 fully rescue phenotypes caused by shRNA-mediated depletion, and replacement transgenes encoding insensitive mutant histone H3.2K27 result in phenotypes associated with Polycomb loss of function upon H3.2 shRNA expression. Thus, this selective depletion system can be used to directly test histone residue function *in vivo*.

Current methods to generate histone mutants in *Drosophila* rely on homozygous deletion of the histone locus coupled with expression of tailor-made transgenic histones [23]. However, many histone mutations are recessive lethal which limits the developmental contexts in which they can be studied. For instance, H3.2K27 mutants die during embryogenesis [13, 14]. These embryos exhibit mild homeotic transformations and Polycomb target gene derepression because most embryonic cells divide a limited number of times after zygotic transcription of H3.2K27 mutant histones initiates, and maternally deposited wild-type histones suppress mutant phenotypes prior to the onset of zygotic transcription. In such circumstances, genetic mosaic analysis via mitotic recombination has been utilized to overcome these limitations. However, only a relatively small number of homozygous mutant cells that are mixed with wild-type cells can be obtained through mitotic recombination, limiting the types of downstream analyses such as biochemical assays or genomic profiling that can be performed. Selective depletion of endogenous H3.2 through RNAi overcomes this shortcoming due to the availability of GAL4 drivers with a wide range of spatiotemporal expression patterns, thus providing the opportunity study histone mutant phenotypes at the level of whole tissues and organs.

One major question regarding the efficacy of an RNAi-based system is whether it would suffice to deplete the massive quantities of histone gene products present in cells. Each S phase, millions of H3 proteins are needed to package the newly replicated genome, raising the possibility that shRNA-mediated knockdown would fail to deplete endogenous histone gene products to sufficiently low levels for a phenotypic consequence. A related potential drawback is whether there would be a delay in knockdown while the shRNA accumulated to functional levels. However, we observed diminished histone H3.2 protein levels near the theoretical maximum within one cell cycle of shRNA expression in embryos (Fig. 2). We also observed rapid loss of shRNA-expressing cells from the wing imaginal disc (Fig. 3E), consistent with many prior studies demonstrating elimination of cells that have a growth disadvantage from a proliferating tissue [49]. Collectively, our findings demonstrate that shRNA-mediated knockdown in proliferating tissues can rapidly overcome the high expression levels of histone H3.2, one of the most abundant gene products in cells. We note, however, that this RNAi-based system would not be appropriate for use in post-replicative cells.

Success of our approach depends on the presence of shared sequence identity such that all histone gene copies can be targeted by the shRNA. Recent assembly of the *D. melanogaster* histone gene locus enabled us to determine that sequences within the 3’-UTR were identical across all histone *H3.2* gene copies. The applicability of this experimental approach to other histone genes, or to mammals and other animal models, would depend on finding similar stretches of high sequence identity across all paralogs. Alternatively, pools of shRNA constructs could be employed to simultaneously knockdown endogenous histone gene expression. If so, selective depletion of endogenous histone gene expression via shRNA would provide a broadly powerful experimental approach for interrogating histone mutant phenotypes.

## Conclusions

We describe here an experimental approach for examining the function of histone post-translational modifications during animal development, building upon the capabilities of existing histone gene replacement methods. We show that transgenic shRNAs are capable of depleting endogenous replication-dependent histone H3.2 despite its exceptional abundance. We demonstrate that this loss of endogenous H3.2 causes defects that are similar to those observed upon mutation of H3.2. Moreover, transgenic wild-type histones engineered to be insensitive to RNAi can rescue the defects caused by shRNA expression. Last, we demonstrate that this experimental platform causes derepression of Polycomb target genes when coupled with an shRNA-insensitive *H3.2*^*K27R*^ transgene. Thus, selective depletion of endogenous histones permits study of the consequences of histone gene mutation during animal development with high spatiotemporal resolution.

## Supplementary Material

Supplementary Files

This is a list of supplementary files associated with this preprint. Click to download.

- SupplementalFigures.docx

## Data Availability

Data and materials will be made available upon request.
